# Dynamics of Wnt/β-catenin reporter activity throughout whole life in a naturally short-lived vertebrate

**DOI:** 10.1038/s41514-024-00149-1

**Published:** 2024-04-29

**Authors:** Shohei Ogamino, Moeko Yamamichi, Ken Sato, Tohru Ishitani

**Affiliations:** 1 Department of Homeostatic Regulation, Division of Cellular and Molecular Biology, Research Institute for Microbial Diseases, Osaka University, Suita, Osaka 565-0871 Japan; 2 Institute for Molecular & Cellular Regulation, Gunma University, Gunma, 371-8512 Japan; 3 Center for Infectious Disease Education and Research (CiDER), Osaka University, Suita, Osaka 565-0871 Japan

**Keywords:** Ageing, Model vertebrates, Model vertebrates

## Abstract

Wnt/β-catenin signaling plays a major role in regulation of embryogenesis, organogenesis, and adult tissue homeostasis and regeneration. However, the roles played by Wnt/β-catenin and the spatiotemporal regulation of its activity throughout life, including during aging, are not fully understood. To address these issues, we introduced a Wnt/β-catenin signaling sensitive reporter into African turquoise killifish (*Nothobranchius furzeri*), a naturally ultra-short-lived fish that allows for the analysis of its whole life within a short period of time. Using this reporter killifish, we unraveled the previously unidentified dynamics of Wnt/β-catenin signaling during development and aging. Using the reporter strain, we detected Wnt/β-catenin activity in actively developing tissues as reported in previous reports, but also observed activation and attenuation of Wnt/β-catenin activity during embryonic reaggregation and diapause, respectively. During the aging process, the reporter was activated in the choroidal layer and liver, but its expression decreased in the kidneys. In addition, the reporter also revealed that aging disrupts the spatial regulation and intensity control of Wnt/β-catenin activity seen during fin regeneration, which interferes with precise regeneration. Thus, the employed reporter killifish is a highly useful model for investigating the dynamics of Wnt/β-catenin signaling during both the developmental and aging process.

## Introduction

The Wnt/β-catenin signaling pathway is an evolutionarily conserved system that controls cell fate determination, proliferation, and cell polarity by increasing β-catenin levels and altering gene expression through Tcf/Lef transcription factors^1^. It also plays an important role in embryogenesis, organogenesis, and adult tissue homeostasis^2^. The role of the Wnt/β-catenin signaling pathway and its spatiotemporal dynamics have been extensively analyzed using model organisms with gene knockout/knockdown and transgenic Wnt/β-catenin signaling reporters^3–11^. However, the long lifespan of these model animals (approximately three years for mice and zebrafish) makes it time-consuming to understand the functions and regulation of Wnt/β-catenin signaling during the vertebrate aging process.

African turquoise killifish (*Nothobranchius furzeri*) is a promising model organism that could be useful for studying aging, age-related diseases, and embryonic diapause^12–14^. The representative inbred *N. furzeri* line, GRZ, has an extremely short lifespan in captivity. GRZ larvae grow rapidly and sexually mature by 4–5 weeks, after which the adult fish age quickly and die approximately within 3–4 months^15,16^, which makes them suitable for the short-term analysis of the vertebrate aging process. Furthermore, *N. furzeri* exhibits similar aging phenomena as observed in humans and mice^17^. In addition to its short lifespan, it has two unique developmental features that are quite different from those of other teleost species, such as zebrafish and medaka. First, its embryos undergo cell dispersion and re-aggregation before gastrulation^18,19^. Second, some of the embryos enter a state of reversible developmental arrest^20^. However, the signaling dynamics of these unique processes remain unclear.

In the present study, we created a new transgenic *N. furzeri* line carrying a Wnt/β-catenin signaling reporter, Tcf/Lef-miniP:dGFP, that emitted clear green fluorescence at various known Wnt/β-catenin signaling active sites in the developing embryonic tissues, organs, and regenerating adult tissues. We revealed the dynamics of Wnt/β-catenin activity during embryonic reaggregation, diapause, and aging in the liver, kidneys, and eyes. In addition, we discovered the involvement of Wnt/β-catenin signaling in the age-dependent decline in regenerative capacity. Thus, this transgenic fish is a useful model for investigating the differences in function and regulation of Wnt/β-catenin signaling throughout life.

## Results

### Generation of transgenic *N. furzeri* Wnt/β-catenin signaling reporter strain

**Fig. 1:**
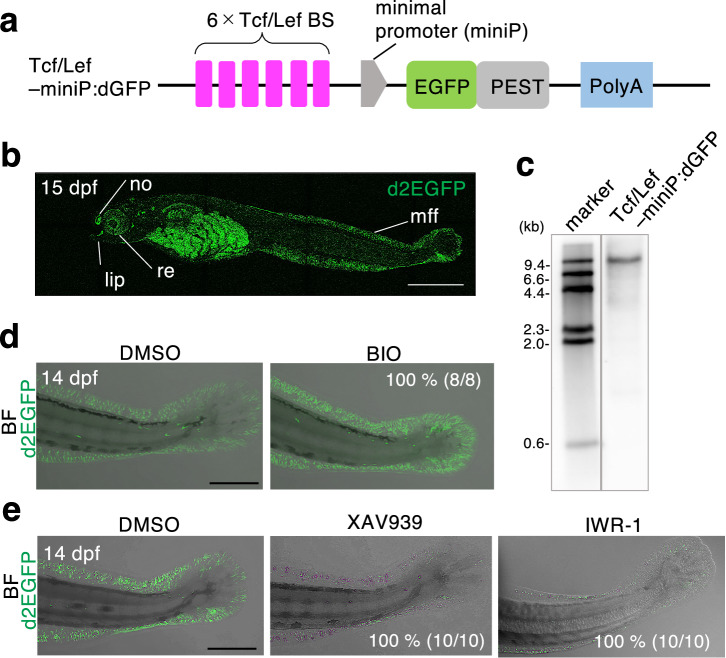
Generation of the Wnt/β-catenin signaling reporter *N. furzeri* strain. **a** Schematic diagram showing the Wnt/β-catenin signaling-reporter constructs. Tcf/Lef BS: consensus sequence of the Tcf/Lef-binding site. PolyA: SV40 polyadenylation sequence. **b** Protein expression of d2EGFP 15 days-post-fertilization (dpf) in the Tcf/Lef-miniP:dGFP transgenic *N. furzeri* strain, anterior to the left. Green fluorescence protein (GFP)-expressing cells were visualized using macro-confocal microscopy. No: nose; re: retina; mff: median fin fold. Scale bar, 200 μm. **c** Southern blot analysis of the transgenes in the Tcf/Lef-miniP:dGFP transgenic *N. furzeri* lines. **d** Tcf/Lef-miniP:dGFP reporter activity was enhanced by 6-bromoindirubin-3-oxim (BIO) treatment. Left-side views of fish treated with 10 μM BIO for 8 h, with the anterior side on the left. Scale bar, 100 μm. **e** XAV939 or IWR-1 treatment reduced Tcf/Lef-miniP:dGFP reporter activity. The panels show the lateral view after treatment with dimethyl sulfoxide, 10 μM XAV939, or IWR-1 for 10 h, with the anterior side to the left. The d2EGFP-expressing cells were visualized using macro-confocal microscopy. The fluorescence images were merged with the bright-field (BF) images. Scale bar, 100 μm.

We previously constructed a Wnt/β-catenin reporter construct, Tcf/Lef-miniP:dGFP, that could efficiently visualize the signaling dynamics in zebrafish embryos^10^. Tcf/Lef-miniP:dGFP contains six Tcf/Lef-binding sites, an artificial synthetic minimal promoter containing TATA boxes (miniP), and destabilized enhanced green fluorescence protein (EGFP; destabilized GFP; Fig. 1a). To visualize the Wnt/β-catenin activity in *N. furzeri*, we introduced this construct into *N. furzeri* embryos via Tol2 transposase-mediated transgenesis^21,22^. Tcf/Lef-miniP:dGFP-transgenic embryos expressed dGFP in the retina, lip, nose, and median fin fold (Fig. 1b) 15 days-post-fertilization (dpf), which have been previously reported as Wnt/β-catenin signaling-active sites^23–25^. We obtained a stable line carrying a single copy of the transgene by outcrossing three times with the wild-type fish (Fig. 1c). To confirm that the reporter reflects Wnt/β-catenin signaling activity, transgenic embryos were treated with chemical Wnt/β-catenin activators and inhibitors. Treatment with a known activator of Wnt/β-catenin signaling^26^, 6-bromoindirubin-3-oxim (BIO), upregulated reporter activity (Fig. 1d), whereas treatment with XAV939 or IWR-1, which are inhibitors^27,28^, decreased dGFP fluorescence (Fig. 1e). Taken together, these data suggest that the Tcf/Lef-miniP:dGFP transgenic *N. Furzeri* stain expresses dGFP in a Wnt/β-catenin signaling-dependent manner.

### Dynamic expression of Tcf/Lef-miniP:dGFP in *N. furzeri* embryos and larvae

**Fig. 2:**
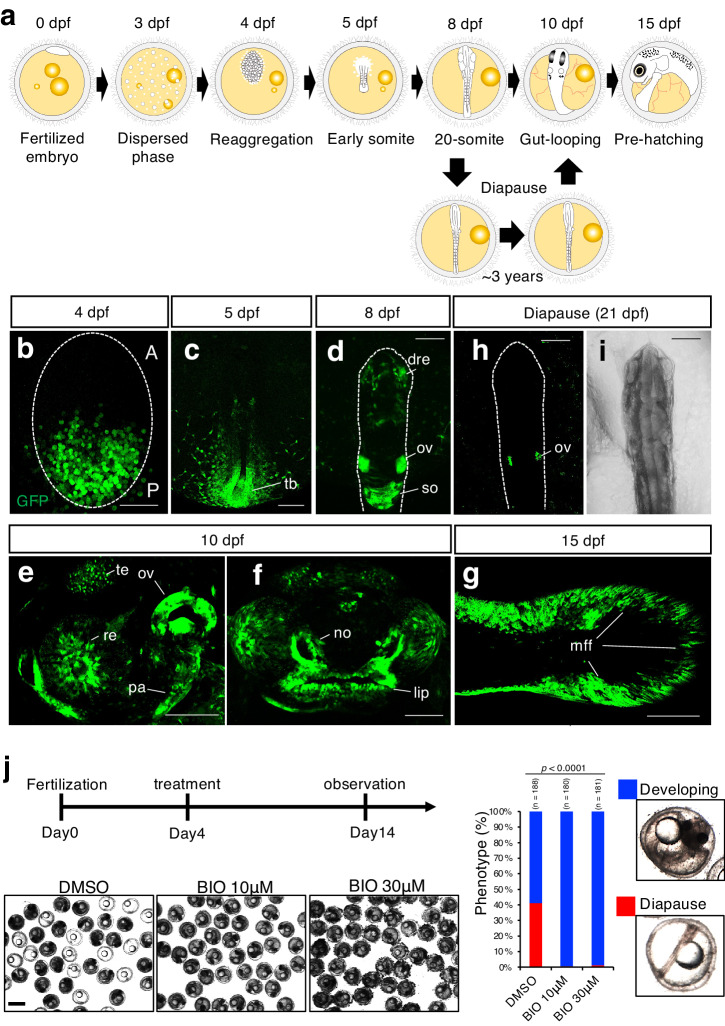
Tcf/Lef-miniP:dGFP reporter activation in *N. furzeri* embryos and larvae. **a** Schematic representation of *N. furzeri* development from the zygote period to prehatching. *N. furzeri* can enter a state of dormancy, termed diapause II, and then restart the development. **b**–**g** GFP expression in Tcf/Lef-miniP:dGFP transgenic *N. furzeri* embryos and larvae. GFP-expressing cells were visualized using macro-confocal microscopy. Scale bar, 100 μm. Dorsal view of 4-(**b**), 5-(**c**) and 8-(**d**) dpf embryos. Tail bud (tb), dorsal retina (dre), and newly formed somites (so). Left-side head and front facial view of 10-dpf embryos, with the dorsal side to the top (**e**, **f**). Tectum (te), retina (re), developing pharyngeal arch (pa), otic vesicles (ov), and nose (no). **g** Left-side tail view of 15-dpf embryos. Median fold change (mff). **h, i** Dorsal view of 21-dpf diapause embryo. Otic vesicle (ov). GFP-expressing cells were visualized using fluorescence microscopy (right panels). The left panel shows a BF image. Scale bar, 200 μm. **j** Images show 14-dpa embryos treated with DMSO or BIO (10 or 30 μM). Percentages of developing or diapause embryos are shown. The numbers shown above the graph indicate the total number of embryos analyzed. Fisher’s exact test was used. Scale bar, 1 mm.

Next, we used this reporter to observe the dynamics of signaling during embryogenesis in live embryos. *N. furzeri* embryos undergo unique developmental processes, including cell dispersion, reaggregation, and diapause (developmental arrest)^18–20^ (Fig. 2a). We first detected reporter activation on the presumptive posterior side of the reaggregation-stage embryos (Fig. 2b). Activation of Wnt/β-catenin signaling in the posterior embryonic tissue plays an essential role in anterior-posterior (AP) axis formation^29^ as this activation regulates the formation of AP axis. At the somite stage, GFP-positive cells were observed at known signaling-active sites seen in other vertebrates (Fig. 2c–g), including the tail bud, newly formed somite, dorsal retina, and posterior lateral line primordium (prim-I)^10,30^ (Fig. 2d). Interestingly, their signaling activities were attenuated in diapause embryos (Fig. 2h, i). Moreover, forced activation of Wnt/β-catenin signaling by BIO treatment severely reduced the number of the diapause-state embryos (Fig. 2j), suggesting that an appropriate reduction in Wnt/β-catenin signaling may be required for entry into the diapause state. From 10 to 15 dpf, fluorescence was detected in other known active sites as well, such as the otic vesicle^31^, developing pharyngeal arch^32^, growing midbrain tectum^10^, retina^23^, lip^24^, and caudal fin mesenchymal cells^25^ (Fig. 2e-g). These data suggest that the employed reporter accurately detects Wnt/β-catenin signaling.

### Wnt/β-catenin activity dynamics during the aging process in *N. furzeri*

**Fig. 3:**
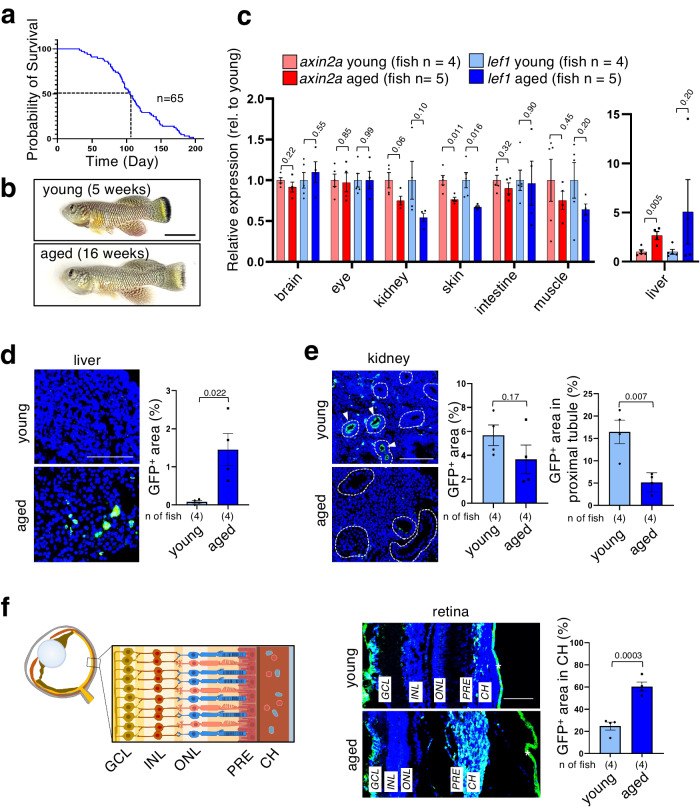
Dynamics of Wnt/β-catenin signaling in young adult and aged *N. furzeri.* **a** Survival curve of *N. furzeri* strain GRZ. The mean lifespan of the wild-derived strain (*n* = 31; housed in single tanks) was 16 weeks, and the maximum lifespan (10% survival) was 20 weeks. Based on the survival curve, two age groups (5 and 16 weeks) were determined for the subsequent experiments. **b** Representative images of young and aged *N. furzeri* males. Scale bar, 10 mm. **c** Quantitative reverse transcription-polymerase chain reaction (qPCR) analysis was performed to determine the gene expression in each age group. Gene expression was normalized to the reference gene expression, *tbp*. Graphs show the percentage of relative expression of *axin2* or *lef1* in each tissue (mean ± SEM). An unpaired two-tailed *t-*test was used for statistical analysis. **d**, **e** Fluorescent images of the indicated organs from young (5 weeks) and aged (16 weeks) Tcf/Lef-miniP:dGFP reporter *N. furzeri*. Scale bar, 100 μm. Graphs show the mean ± SEM of the GFP-positive (GFP^+^) areas in the liver and kidneys. An unpaired two-tailed *t-*test was used for statistical analysis. **f** Fluorescent images of the eyes of young (5 weeks) and aged (16 weeks) Tcf/Lef-miniP:dGFP reporter *N. furzeri*. Scale bar, 100 μm. Ganglion cell layer (GCL), nerve fiber layer (NFL), inner plexiform layer (IPL), inner nuclear layer (INL), outer plexiform layer (OPL), outer nuclear layer (ONL), photoreceptor segment layer (PRL), and choroid (CH). Graphs show the mean ± SD of GFP-positive areas in the retinal choroid. **d**–**f** An unpaired two-tailed *t-*test was used for statistical analysis.

We analyzed Wnt/β-catenin signaling in young and aged *N. furzeri* to explore age-dependent changes in Wnt/β-catenin signaling activity. We considered 16-week-old fish as “aged,” as the median lifespan of *N. furzeri* was 16 weeks in our system (Fig. 3a). We observed aging-associated phenotypes, such as emaciation and color loss in aged fish (Fig. 3b). First, we performed quantitative reverse transcription-PCR (qPCR) analysis for *axin2* and *lef1*, which are the known target genes of the Wnt/β-catenin signaling pathway. Their expression was elevated in the livers of aged fish as compared with that of the livers of young fish and was significantly downregulated in the skin of aged fish. Their expression tended to decrease in muscles and kidneys of aged fish (Fig. 3c). Next, we attempted to detect aging-related spatial changes in Wnt/β-catenin signaling activity using the *N. furzeri* Tcf/Lef-miniP:dGFP reporter strain. Consistent with the qPCR results, GFP-positive cells appeared and increased significantly in aged fish livers compared to young livers (Fig. 3d). In contrast, kidneys of aged fish tended to have reduced GFP-positive areas compared with the kidneys of young fish (Fig. 3e). This GFP-downregulated area seemed to be the proximal tubules (Supplementary Fig. 1). Abnormal activation of Wnt/β-catenin signaling promotes carcinogenesis and fibrosis in the liver^33,34^ and the decrease in Wnt/β-catenin signaling caused epithelial injury and renal fibrosis in the kidney proximal tubules^35^. Therefore, an increase in Wnt/β-catenin activity in the liver and a decreased activity in the kidney may contribute to age-associated impairment of these organs. We also detected age-associated activation of Wnt/β-catenin signaling in the choroidal layer of eyes in aged fish (Fig. 3f; Supplementary Fig. 1), although qRT-PCR analysis showed no significant difference between the eyes of young and aged fish (Fig. 3c). Although the role of age-associated Wnt/β-catenin activation in the choroid remains unclear, this activation may be involved in aging-related eye diseases such as age-related macular degeneration (AMD).

### Impaired Wnt/β-catenin signal activity causes age-dependent decline in regeneration capability

**Fig. 4:**
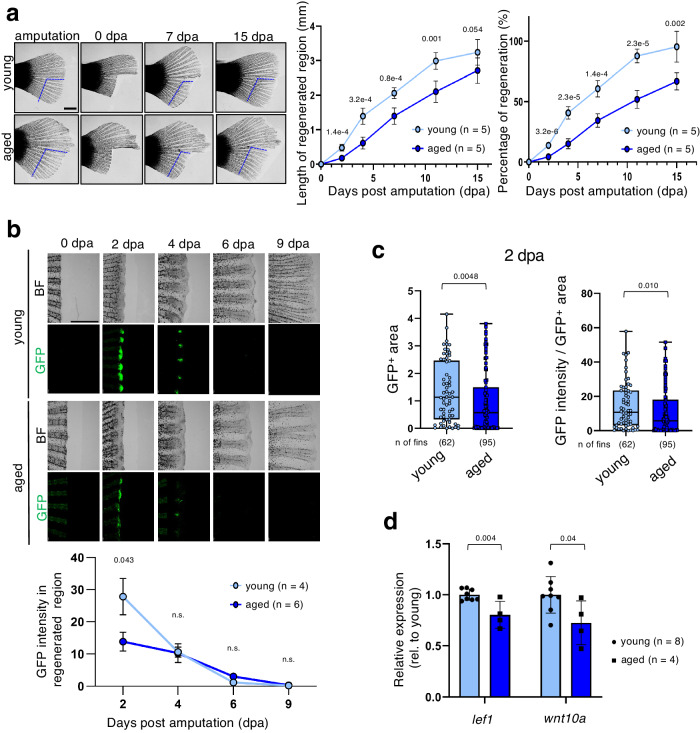
Age-dependent reduction in Wnt/β-catenin activity during amputated fin regeneration. **a** Caudal fins from young and aged fish were amputated at the indicated blue lines, as shown for 15 days [0–15 days post amputation (dpa)]. Scale bar, 1 mm. Graphs show the means ± SEM of the length of the regenerated region and the percentage of regeneration (length of regenerated region/length of amputated region) in regenerating caudal fins. **b** Comparison of the Wnt/β-catenin activity in regenerating fins between young and aged fish from 0 to 9 dpa. GFP-expressing cells were visualized using a fluorescence microscope (lower panels). The upper panels show BF images. The graph shows mean ± SEM of the GFP signal intensity of the regenerating caudal fins. An unpaired two-tailed *t-*test was used for statistical analysis. **c** Comparison of GFP-positive (GFP^+^) areas and GFP intensity/GFP^+^ areas in regenerating fins between young and aged fish. The box plot shows the first and third quartiles; the median is represented by a line, and the whiskers indicate the minimum and maximum values. The numbers below the graph indicate the total numbers of fins analyzed. The Mann-Whitney *U* test was used. **d** qPCR analysis was performed to determine the gene expression in regenerating regions at 2 dpa in each age group. Gene expression was normalized to the expression of the reference gene *tbp*. Graphs show the relative expression of *lef1* or *wnt10a* (mean ± SD). An unpaired two-tailed *t-*test was used for statistical analysis.

We investigated the relationship between Wnt/β-catenin signaling and the age-dependent decline in the regeneration potential, as the Wnt/β-catenin signaling regulates adult tissue regeneration^36,37^. It is well established that Wnt/β-catenin signaling is activated in amputated zebrafish fins and contributes to their regeneration by promoting the formation of blastema, which serves as the source of progenitor cells^38^. Recent studies have shown that the fin regeneration capacity of *N. furzeri* declines in an age-dependent manner^39,40^. We observed that the length of the regenerated region and the percentage of regeneration in fins of aged fish were significantly lower than those in fins of young fish (Fig. 4a). The rate of fin regeneration per day in young fish was higher than that in aged fish, especially 4 days post amputation (Supplementary Fig. 2). These results suggest that aging resulted in a decline in fin regeneration. Therefore, we next investigated age-dependent changes in Wnt/β-catenin signaling in fin regeneration. Activation of the Wnt/β-catenin signaling reporter was detected in the regenerating region from 2 days post amputation onward (2 dpa) in both young and aged fish (Fig. 4b). However, the signaling activity in aged fish was significantly lower than that of young fish (Fig. 4b). The peak of Wnt/β-catenin activation was at 2 dpa in both young and aged fish (Fig. 4b), indicating that the difference in Wnt/β-catenin activation levels between young and aged fish does not appear to be due to a delayed activation in aged fish. In addition, we found that the GFP-positive (Wnt/β-catenin reporter-active) area in aged regenerating fins was significantly narrower than that in young regenerating fins (Fig. 4c, left graph). Furthermore, the intensity of GFP in the GFP-positive area decreased with aging as well (Fig. 4c, right graph). These results suggest that aging causes a decrease in both the number of Wnt/β-catenin-active cells and in the activity of Wnt/β-catenin signaling. Consistent with these results, the expression levels of the endogenous Wnt/β-catenin target gene *lef1*, as well as those of *wnt10a*, which is known to be upregulated during fin regeneration and is required for activation of Wnt/β-catenin signaling in blastema formation^41^, were higher in the regenerating fins of young fish than in those of aged fish (Fig. 4d). These results suggest that an age-dependent reduction in Wnt/β-catenin signaling activity may cause the decline in fin regeneration.

**Fig. 5:**
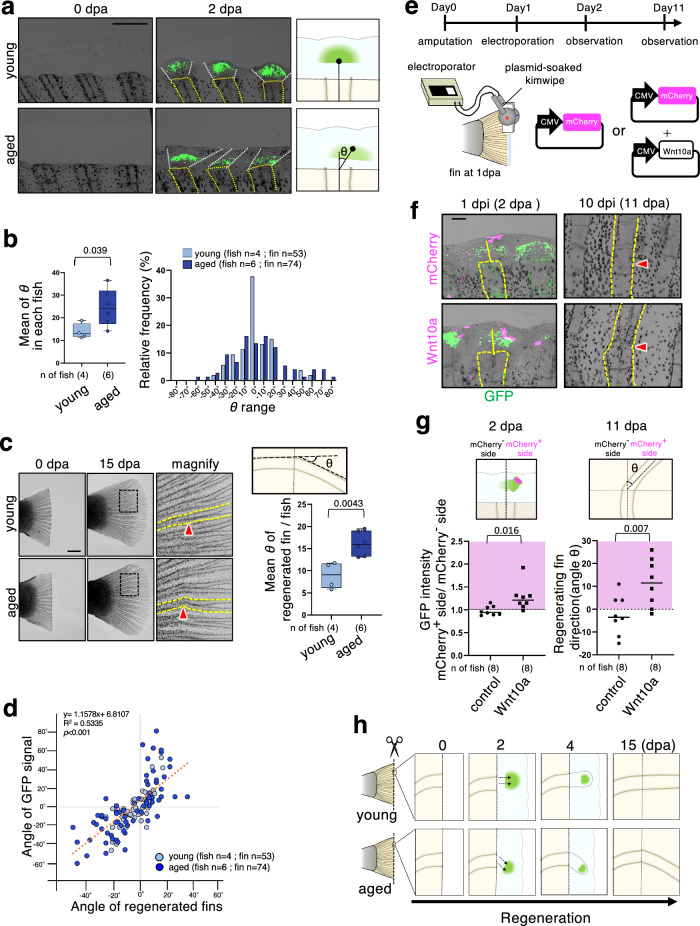
Age-associated changes in the spatial regulation of Wnt/β-catenin activity may be involved in the decline of the regeneration ability. **a,** **b** Comparison of Wnt/β-catenin activity in regenerating fins between young and aged fish from 0 to 2 dpa. **a** GFP-expressing cells were visualized using a fluorescence microscope. The fluorescence images were merged with the bright-field (BF) images. The bones in the fin rays are outlined with yellow dotted lines. The GFP^+^ area is outlined with white dotted lines. Schematic illustrations show the GFP expression pattern in regenerating fins of young and aged fish. *θ* indicates the angle between an extension of the bone centerline and a line connecting the center point of the GFP^+^ area and that of the cutting edge of the bone. **b** The left graph shows the mean *θ* in each fish at 2 dpa. The box plot shows the first and third quartiles; the median is represented by a line, and the whiskers indicate the minimum and maximum values. The right graph shows a relative frequency histogram of *θ* at 2 dpa. **c** Microscopic images of the amputated (0 dpa) and regenerating caudal fins (15 dpa). The yellow dotted lines indicate the fin rays’ bone. The graph shows the mean angle (*θ*) of regenerated fins per fish at 15 dpa. The box plot shows the first and third quartiles; the median is represented by a line, and the whiskers indicate the minimum and maximum values. Red arrowheads indicate sites of amputation. **d** Correlation analysis of the angle of the GFP signal relative to the bone direction and angle of the subsequently regenerated fin. Pearson product-moment correlation coefficient (PPMCC) was used. **e** Schematic illustration of the experimental system for artificially activating Wnt/β-catenin signaling in regenerating fins of *N. furzeri*. Kimwipe soaked in 1 μg/μl DNA solution [CMV promoter-driven expression vector (pCS2p+) carrying mCherry or Wnt10a or an equal amount of empty vector] was wrapped around the fins and electroporated using platinum electrodes. The upper illustration shows the experimental timeline. **f** Microscopic images showing GFP-expressing cells (GFP) and introduced mCherry-expressing cells (magenta) in regenerating fins at 2 and 11 dpa. The fluorescence images were merged with the BF images. Red arrowheads indicate sites of amputation. **g** The left graph shows the GFP intensity (Wnt/β-catenin reporter activity) in the mCherry-positive (mCherry^+^) side vs. that in the mCherry-negative (mCherry^-^) side at 2 dpa. Each dot represents one fin. The right graph shows the regenerating fin direction at 11 dpa. Each dot represents one fin. **h** Schematic illustration of age-associated mispositioning signal activation that promoted misdirected fin regeneration. **b, c, g** An unpaired two-tailed *t-*test was used. **a,** **f** Scale bar, 200 μm. **c** Scale bar, 500 μm.

Notably, in addition to the reduction of Wnt/β-catenin activity, mispositioning of Wnt/β-catenin activation was also detected in regenerating fins of aged fish. In young fish, Wnt/β-catenin signaling was activated at positions along the direction of the bone, but in aged fish, signaling was activated at positions laterally away from the direction of the bone (Fig. 5a, b). The subsequently regenerated fins grew at a misaligned angle (Fig. 5c, Supplementary Fig. 3), which was tightly correlated with the mispositioning of the Wnt/β-catenin signaling activity at 2 dpa (Fig. 5d). Given that Wnt/β-catenin signaling promotes blastema formation, mispositioning of this signaling can cause fins to regenerate in an incorrect direction. To test this idea, we established a system for artificially activating Wnt/β-catenin signaling in regenerating fins of *N. furzeri* by introducing plasmid DNA encoding *wnt10a* (Fig. 5e). As shown in Fig. 5f and g, artificial expression of Wnt10a induced mispositioned activation of Wnt/β-catenin signaling, leading to misaligned rays. These results indicate that mispositioning of Wnt/β-catenin activation leads to misaligned rays and also suggest that an age-dependent change in the positional information of the Wnt/β-catenin signal may be involved in the age-related decline in the regenerating ability (Fig. 5h).

## Discussion

In the present study, we developed a reporter strain that could be used to easily and rapidly investigate the dynamics of Wnt/β-catenin signaling throughout the lifespan of the investigated vertebrate. This reporter detected the signaling sites known to be active in other vertebrates, therefore, confirming that the reporter reliably reflects signaling activity. Using this reporter, we identified previously unidentified dynamics of Wnt/β-catenin signaling in early embryogenesis, diapause, regeneration, and aging.

Previous studies reported age-dependent increases in Wnt/β-catenin signaling in the mouse liver^42^ and an age-dependent decline in Wnt/β-catenin signaling in the human skin^43^. Consistent with these observations, our qPCR analyses revealed that Wnt/β-catenin signaling activity was upregulated in the liver and downregulated in the skin with age in *N. furzeri*. Using the Wnt reporter-transgenic *N. furzeri*, we also succeeded in detecting age-dependent spatial changes in the Wnt/β-catenin activity in the liver, kidneys, and eyes. In the livers of aged fish, abnormal activation of Wnt/β-catenin signaling was detected. There is growing evidence that aberrantly hyperactivated Wnt/β-catenin signaling promotes liver fibrosis^44,45^ and tumorigenesis and progression of hepatocellular carcinoma, which is the most common primary liver cancer that frequently develops in chronic liver disease^46^. Therefore, increased signal intensity can threaten liver function. In the kidneys, an age-dependent decline in Wnt/β-catenin signaling activity was observed in the renal tubular epithelium. Previous studies have revealed that activation of Wnt signaling in the proximal tubules inhibits apoptosis upon injury and promotes the restoration of kidney structure, function, and cell proliferation^47,48^. This indicates that the age-dependent decline in Wnt/β-catenin activity may accelerate epithelial damage, leading to an age-related decline in renal function. Wnt/β-catenin signaling is activated in the choroid in aged fish, which supplies oxygen and nutrients to the retinal pigment epithelium and the photoreceptor cells and helps remove waste products^49^. A decline in one of these functions is a major factor in AMD; thus, investigating the role of age-related changes in Wnt/β-catenin signal activation may be useful in AMD treatment. Taken together, our findings indicate that the Tcf-miniP:dGFP-transgenic *N. furzeri* strain is a useful tool for aging studies, and further analysis of this reporter could help in detailed mechanistic exploration of age-dependent tissue degeneration to establish therapeutic strategies.

Tissue regeneration is regulated by Wnt/β-catenin signaling^36,37^. However, the effects of aging on this activity remain unclear. In this study, we showed that Wnt/β-catenin signaling is activated after fin amputation in *N. furzeri*, similar to that in zebrafish^10,38^, and that this activation is attenuated with aging. In addition, by taking advantage of this reporter’s capability, we succeeded in revealing age-associated ectopic activation of Wnt/β-catenin signaling during regeneration, leading to ectopic formation of the blastema and resulting in formation of misaligned fin rays. These results suggest that the age-related impairment of the regenerative capacity and disruption of morphology is dependent on age-associated changes in the strength and spatial regulation of Wnt/β-catenin activity. However, it remains unclear why aging causes changes in Wnt/β-catenin signaling. Previous studies have shown that forced denervation of fins cause a decline in zebrafish fin regeneration capacity and misaligned fin rays via reduction in Wnt/β-catenin signaling^50^. Therefore, it would be interesting to study the effects of age-associated changes in the nervous system on Wnt/β-catenin signaling.

*N. furzeri* has two unique developmental stages: the “disperse and reaggregation stage” and the “diapause stage.” These stages are only present in annual killifish, including *Nothobranchius* and *Austrofundulu*^19^. However, the involvement of Wnt/β-catenin signaling in these processes remains to be elucidated. Interestingly, our observations at the reaggregation stage showed that Wnt/β-catenin signaling gradients were formed from the posterior side of the embryos. In vertebrate embryos, Wnt/β-catenin signaling is activated in the presumptive posterior tissue, thereby establishing embryonic AP patterning^51^. Wnt/β-catenin activation at the reaggregation stage may establish the AP pattern in *N. furzeri* embryos. We also showed that diapause embryos attenuated Wnt/β-catenin activity, and forced activation of Wnt/β-catenin signaling prevented entry into the diapause state. This suggests that diapause embryos may actively stop Wnt/β-catenin signaling to temporarily arrest development. It will be interesting to focus on Wnt/β-catenin signaling attenuation when studying developmental diapause mechanisms. Thus, these data indicate that this reporter strain could be used to investigate not only aging but also unique developmental processes in annual killifish.

In conclusion, we have shown that our newly generated Tcf/Lef-miniP:dGFP-transgenic *N. furzeri* strain can be a useful model to understand the relationship of Wnt/β-catenin signaling with embryogenesis, aging, and aging-associated diseases. Our findings obtained using this reporter strain may be applicable to humans, as the homologous organs are conserved between killifish and humans^17^.

## Methods

### Fish strain, husbandry, and maintenance

The GRZ (GRZ-AD) strain of *N. furzeri* was a gift from Professor Adam Antebi (Max Plank Institute for the Biology of Aging). Fish were maintained at 26.5 °C, 0.7 conductivity on 12-h/12-h light/dark cycle in the fish breeding system (MEITO, Nagoya, Japan) at a density of one fish per 1.4 L tank for the adult fish. They were fed freshly hatched brine shrimp twice a day from Monday to Saturday and once a day on Sunday; fish that were over 2 weeks old were also fed bloodworms (Kyorin, Himeji, Japan). For mating, one adult male and three to four female fish were kept in 4-L tanks, and the females spawned on a sand substrate in plastic cases. The embryos were collected and incubated in the egg water (0.03% sea salt with methylene blue). Ten days later, the embryos were plated on sterile dry peat moss until they were ready to hatch. Usually, one month after egg collection, the embryos were ready to hatch and incubated in 0.07% ice-cold humic acid solution (Cat no. 53680, Sigma-Aldrich, St. Louis, MO, USA) for 30–60 min and transferred into a 4-L hatching tank with air supply. Hatched fry was kept in the hatching tank, and half of the solution was replaced with fish breeding system water daily. After two weeks, when the fish had grown to ~10–15 mm, they were transferred to 1.4-L tanks, similar to the adult fish. Fish for the experiments were euthanized by immersion in MS222 (Sigma-Aldrich, St. Louis, MO, USA) solution (400 mg/L). All experimental animal care was performed in accordance with the institutional and national guidelines and regulations. The study protocol was approved by the Institutional Animal Care and Use Committee of Osaka University (RIMD Permit# R02-04). The study was conducted in accordance with the ARRIVE guidelines.

### mRNA synthesis

Tol2 transposase mRNA was generated using the pCS-TP plasmid^21^ as a template. Capped mRNA was synthesized using the SP6 mMessage mMachine kit (Ambion, Austin, TX, USA) and purified using Micro Bio-Spin columns (Bio-Rad, Hercules, CA, USA).

### Generation of transgenic *N. furzeri*

We co-injected 25–50 pg of the reporter plasmid (Tcf/Lef-miniP:dGFP), which was previously established in our laboratory, with 30–40 pg of Tol2 transposase mRNA into the one-cell-stage GRZ strain embryos. Transgenic fish strongly expressing d2EGFP were outcrossed with wild-type fish to produce the founder line, Tg(Tcf/Lef-miniP:dGFP), which was maintained by d2EGFP-positive sibling intercrossing.

### Genomic DNA isolation and Southern blot analysis

The tail fins of adult transgenic fish were amputated using a razor edge and then transferred to lysis buffer containing 0.1 μg/μl Proteinase K. The sample was incubated at 55 °C overnight, followed by standard ethanol precipitation. Purified genomic DNA samples were digested with EcoRl, which cleaves the plasmid reporter once. Southern blot hybridization was performed using a digoxigenin-labeled probe (Roche Diagnostics, Basel, Switzerland) and standard methods. Uncropped scan of Southern blot is shown in Supplementary Fig. 4.

### Chemical inhibitor treatment

BIO (Wako, Osaka, Japan), XAV939, and IWR-1 (Enzo Life Sciences, Farmingdale, NY, USA) were dissolved in dimethyl sulfoxide (DMSO). The embryos were treated with 10 μM BIO, XAV939, IWR-1, or 1% DMSO control in the dark at 28.5 °C.

### *N. furzeri* lifespan measurement

The housing conditions were the same as those described above. The fish were fed freshly hatched brine shrimp twice daily from Monday to Saturday and once daily on Sunday. They were also fed bloodworms twice daily from two weeks of age. Fish mortality was documented daily from the age of one month. Lifespan analyses were performed using GraphPad Prism (GraphPad Software, San Diego, CA, USA) for survival curves using the Kaplan–Meier estimator.

### qPCR

Total RNA from the tissues was purified using TRIzol reagent (Invitrogen), and cDNA was synthesized using ReverTra Ace qPCR RT Master Mix (Toyobo, Osaka, Japan). The qPCR analysis of DNA was performed using THUNDERBIRD SYBR qPCR Mix (Toyobo) and *axin2* primers [forward (FW) primer: CATATCGCAGCATGATGAAG, reverse (RV) primer: CTCGCCTTCTTGAAGTAG)], *lef1* primers (FW primer: CCCCAGCTACCCAAGTTACA, RV primer: GTGGTGTGAGAGGGTGGACT), and *wnt10a* primers (FW primer: CCAGCCTTGAGACGAGAAAC, RV primer: CGTACGCAAAGGCACTCTCT) on an Applied Biosystems 7500 real-time PCR system (Applied Biosystems, Carlsbad, CA, USA). *tbp* (FW primer: CGGTTGGAGGGTTTAGTCCT; RV primer: GCAAGACGATTCTGGGTTTG) was used as the loading control for real-time PCR. The analysis was performed in biological replicates [Fig. 2c: *n* = 4 fish in the young (5 weeks) group, *n* = 5 fish in the aged (16 weeks) group, Fig. 4d: *n* = 8 fish in the young (5 weeks) group, *n* = 4 fish in the aged (16 weeks) group].

### Immunostaining and imaging

Tissues were fixed with 4% paraformaldehyde in phosphate-buffered saline (PBS) overnight at 4 °C. They were placed in 10 and 20% sucrose/PBS until the tissues sank, and 30% sucrose/PBS overnight at 4 °C. The tissues were embedded in Tissue-Tek OCT freezing medium (Sakura Finetek, Tokyo, Japan). Slices (10-μm thick) were prepared using Thermo fisher HM525NX cryostat and stored at −80 °C until required. The sections were washed with 0.5% Triton X-100 (PBST) four times and blocked with 10% fetal bovine serum, 4% Block Ace (Megmilk Snow Brand, Tokyo, Japan), and 1% DMSO in 0.1% PBST for 1 h. The sections were then incubated with the primary antibody, anti-GFP (#A-11122, Invitrogen, 1/500), overnight at 4 °C, and then washed and incubated with secondary antibody, AlexaFluor647-conjugated anti-rabbit IgG (#A-32733, Invitrogen, 1/500), and with Hoechst33342 (Invitrogen) overnight at 4 °C. Images were acquired with FV3000 (Olympus, Tokyo, Japan). To measure the GFP-positive area without bias by regions, multiple sections were acquired randomly in the liver and kidney, and images of these entire sections were measured. In the eye, the central retina at 200 µm from the optic nerve head was measured. Quantification was performed using the ImageJ software.

### Hematoxylin and eosin staining

Tissues were fixed with 4% paraformaldehyde in phosphate-buffered saline (PBS) overnight at 4 °C. They were placed in 10 and 20% sucrose/PBS until the tissues sank, and 30% sucrose/PBS overnight at 4 °C. The tissues were embedded in Tissue-Tek OCT freezing medium (Sakura Finetek, Tokyo, Japan). Slices (10-μm thick) were prepared using Thermo Fisher HM525NX cryostat and stored at −80 °C until required. Sections of 14 μm thickness were stained with hematoxylin and eosin according to a standard procedure.

### Plasmids

To generate plasmids expressing Wnt10a proteins, the PCR-amplified cDNA encoding *wnt10a* gene was cloned into the multi-cloning site of the pCS2p+ vector using the In-Fusion® HD Cloning Kit (Takara, Kusatsu, Japan).

### Electroporation

Kimwipe (Kimberly–Clarke, Roswell, GA, USA) soaked in 1 μg/μl DNA solution was wrapped around the fins and electroporated using platinum electrodes. Electroporation was performed using NEPA21 Type II (NEPA GENE Co. Ltd., Chiba, Japan), generating two types of square pulses, namely poring and transfer pulses. The electroporation parameters were as follows; poring pulse: 50 V, 30 ms pulse, 50 ms pulse interval, number of pulses: two, 10% decay (± pulse orientation) and transfer pulse: 30 V, 50 ms pulse, 50 ms pulse, number of pulses: three, 40% decay (±pulse orientation).

### Imaging

Bright-field and fluorescent images of the amputated fins were captured using an M205FA fluorescence stereomicroscope (Leica, Wetzlar, Germany). Fluorescence images of immunostained sections were captured using an FV 3000 confocal laser scanning microscope (Olympus, Tokyo, Japan). The collected image data were processed using the ImageJ software.

### Statistical analysis

Differences between the groups were examined using a two-tailed unpaired Student *t* test, one-way ANOVA, and Fisher’s exact test in Excel (Microsoft, Redmond, WA) or GraphPad Prism 8 (GraphPad Software, San Diego, CA). A *p* value < 0.05 was considered statistically significant.

### Reporting summary

Further information on research design is available in the Nature Research Reporting Summary linked to this article.

### Supplementary information

Reporting Summary

Supplementary Figures

## Data Availability

All the data supporting this study are available from the corresponding author, T. I., upon reasonable request.

## References

[1] Lien WH, Fuchs E (2014). Wnt some lose some: transcriptional governance of stem cells by Wnt/beta-catenin signaling. Genes Dev..

[2] Steinhart Z, Angers S (2018). Wnt signaling in development and tissue homeostasis. Development.

[3] Liu P (1999). Requirement for Wnt3 in vertebrate axis formation. Nat Genet..

[4] Korinek V (1997). Constitutive transcriptional activation by a beta-catenin-Tcf complex in APC-/- colon carcinoma. Science.

[5] DasGupta R, Fuchs E (1999). Multiple roles for activated LEF/TCF transcription complexes during hair follicle development and differentiation. Development.

[6] Lustig B (2002). Negative feedback loop of Wnt signaling through upregulation of conductin/axin2 in colorectal and liver tumors. Mol. Cell Biol.

[7] Maretto S (2003). Mapping Wnt/beta-catenin signaling during mouse development and in colorectal tumors. Proc. Natl Acad. Sci. USA.

[8] Moro E (2012). In vivo Wnt signaling tracing through a transgenic biosensor fish reveals novel activity domains. Dev. Biol.

[9] Mohamed OA, Clarke HJ, Dufort D (2004). Beta-catenin signaling marks the prospective site of primitive streak formation in the mouse embryo. Dev. Dyn..

[10] Shimizu N, Kawakami K, Ishitani T (2012). Visualization and exploration of Tcf/Lef function using a highly responsive Wnt/beta-catenin signaling-reporter transgenic zebrafish. Dev. Biol..

[11] Takemoto T (2016). R26-WntVis reporter mice showing graded response to Wnt signal levels. Genes Cells.

[12] Platzer M, Englert C (2016). Nothobranchius furzeri: a model for aging research and more. Trends Genet..

[13] Terzibasi Tozzini E, Cellerino A (2020). Nothobranchius annual killifishes. Evodevo.

[14] Hu CK, Brunet A (2018). The African turquoise killifish: a research organism to study vertebrate aging and diapause. Aging Cell.

[15] Genade T (2005). Annual fishes of the genus Nothobranchius as a model system for aging research. Aging Cell.

[16] Kirschner J (2012). Mapping of quantitative trait loci controlling lifespan in the short-lived fish Nothobranchius furzeri–a new vertebrate model for age research. Aging Cell.

[17] Kim Y, Nam HG, Valenzano DR (2016). The short-lived African turquoise killifish: an emerging experimental model for ageing. Dis. Model Mech..

[18] Dolfi L, Ripa R, Cellerino A (2014). Transition to annual life history coincides with reduction in cell cycle speed during early cleavage in three independent clades of annual killifish. Evodevo.

[19] Podrabsky JE (2017). Embryonic development of the annual killifish Austrofundulus limnaeus: an emerging model for ecological and evolutionary developmental biology research and instruction. Dev. Dyn..

[20] Hu CK (2020). Vertebrate diapause preserves organisms long-term through Polycomb complex members. Science.

[21] Kawakami K (2004). A transposon-mediated gene trap approach identifies developmentally regulated genes in zebrafish. Dev. Cell.

[22] Urasaki A, Morvan G, Kawakami K (2006). Functional dissection of the Tol2 transposable element identified the minimal cis-sequence and a highly repetitive sequence in the subterminal region essential for transposition. Genetics.

[23] Yamaguchi M (2005). Histone deacetylase 1 regulates retinal neurogenesis in zebrafish by suppressing Wnt and Notch signaling pathways. Development.

[24] Song L (2009). Lrp6-mediated canonical Wnt signaling is required for lip formation and fusion. Development.

[25] Nagayoshi S (2008). Insertional mutagenesis by the Tol2 transposon-mediated enhancer trap approach generated mutations in two developmental genes: tcf7 and synembryn-like. Development.

[26] Meijer L (2003). GSK-3-selective inhibitors derived from Tyrian purple indirubins. Chem. Biol..

[27] Huang SM (2009). Tankyrase inhibition stabilizes axin and antagonizes Wnt signalling. Nature.

[28] Chen B (2009). Small molecule-mediated disruption of Wnt-dependent signaling in tissue regeneration and cancer. Nat. Chem. Biol..

[29] Thorpe CJ, Weidinger G, Moon RT (2005). Wnt/beta-catenin regulation of the Sp1-related transcription factor sp5l promotes tail development in zebrafish. Development.

[30] Ikeya M, Takada S (1998). Wnt signaling from the dorsal neural tube is required for the formation of the medial dermomyotome. Development.

[31] Riccomagno MM, Takada S, Epstein DJ (2005). Wnt-dependent regulation of inner ear morphogenesis is balanced by the opposing and supporting roles of Shh. Genes Dev..

[32] Lewis JL (2004). Reiterated Wnt signaling during zebrafish neural crest development. Development.

[33] Bengochea A (2008). Common dysregulation of Wnt/Frizzled receptor elements in human hepatocellular carcinoma. Br J Cancer.

[34] Marquardt JU (2014). Sequential transcriptome analysis of human liver cancer indicates late stage acquisition of malignant traits. J. Hepatol..

[35] Nlandu-Khodo S (2017). Blocking TGF-β and β-catenin epithelial crosstalk exacerbates CKD. J. Am. Soc. Nephrol..

[36] Clevers H, Loh KM, Nusse R (2014). Stem cell signaling. An integral program for tissue renewal and regeneration: Wnt signaling and stem cell control. Science.

[37] Majidinia M, Aghazadeh J, Jahanban-Esfahlani R, Yousefi B (2018). The roles of Wnt/beta-catenin pathway in tissue development and regenerative medicine. J. Cell Physiol..

[38] Wehner D (2014). Wnt/beta-catenin signaling defines organizing centers that orchestrate growth and differentiation of the regenerating zebrafish caudal fin. Cell Rep..

[39] Wendler S, Hartmann N, Hoppe B, Englert C (2015). Age-dependent decline in fin regenerative capacity in the short-lived fish Nothobranchius furzeri. Aging Cell.

[40] Örling J, Kosonen K, Villman J, Reichard M, Paatero I (2023). Impaired fin regeneration and angiogenesis in aged zebrafish and turquoise killifish. Biol. Open.

[41] Stoick-Cooper CL (2007). Distinct Wnt signaling pathways have opposing roles in appendage regeneration. Development.

[42] Hu HH, Cao G, Wu XQ, Vaziri ND, Zhao YY (2020). Wnt signaling pathway in aging-related tissue fibrosis and therapies. Ageing Res. Rev..

[43] Makrantonaki E (2012). Identification of biomarkers of human skin ageing in both genders. Wnt signalling—a label of skin ageing?. PLoS One.

[44] Monga S (2015). P. beta-catenin signaling and roles in liver homeostasis, injury, and tumorigenesis. Gastroenterology.

[45] Takahara Y (2008). Serial changes in expression of functionally clustered genes in progression of liver fibrosis in hepatitis C patients. World J. Gastroenterol..

[46] Perugorria MJ (2019). Wnt-beta-catenin signalling in liver development, health and disease. Nat. Rev. Gastroenterol. Hepatol..

[47] Terada Y (2003). Expression and function of the developmental gene Wnt-4 during experimental acute renal failure in rats. J. Am. Soc. Nephrol..

[48] Zhou D (2012). Tubule-specific ablation of endogenous beta-catenin aggravates acute kidney injury in mice. Kidney Int..

[49] Chirco KR, Sohn EH, Stone EM, Tucker BA, Mullins RF (2017). Structural and molecular changes in the aging choroid: implications for age-related macular degeneration. Eye.

[50] Simoes MG (2014). Denervation impairs regeneration of amputated zebrafish fins. BMC Dev. Biol..

[51] Petersen CP, Reddien PW (2009). Wnt signaling and the polarity of the primary body axis. Cell.

