# Supplementary material for: Dynamics of Wnt/β-catenin reporter activity throughout whole life in a naturally short-lived vertebrate
Source: NPJ Aging. 2024 Apr 29;10(1):23. doi: 10.1038/s41514-024-00149-1 (PMC11059364; doi:10.1038/s41514-024-00149-1)
Supplement: Supplementary file 2 — Supplementary Figures [file 41514_2024_149_MOESM2_ESM.pdf]

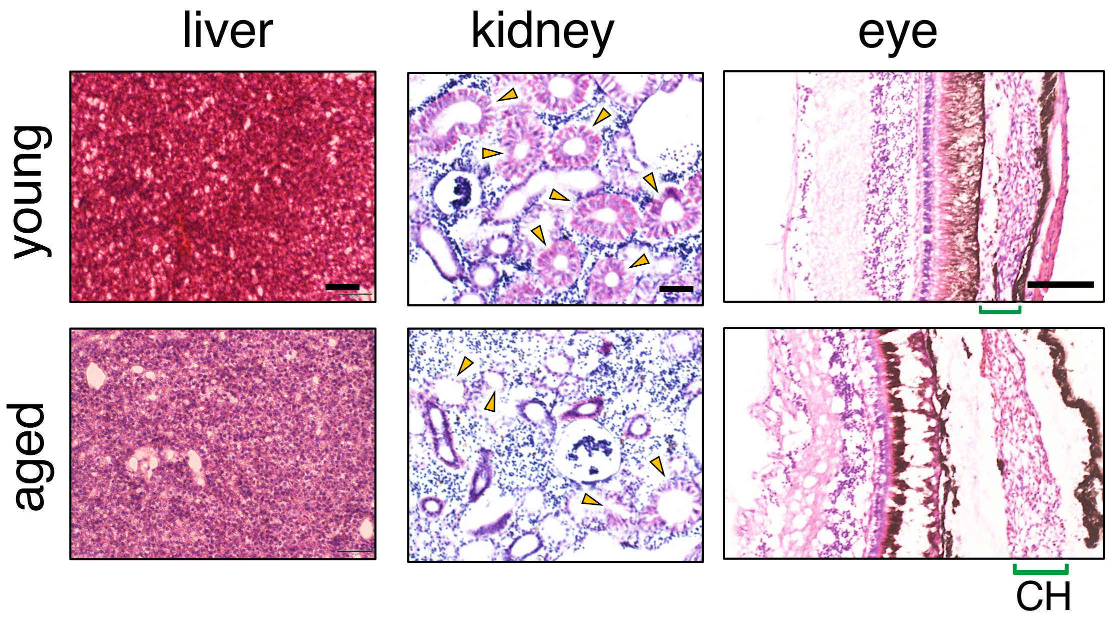

Supplementary Fig. 1: Estimation of Wnt/β-catenin signal activation site using HE staining. Representative image of hematoxylin and eosin staining of the liver, kidney, and eye in each age group. Scale bar: 100 µm. Yellow arrowheads indicated the proximal tubule. Green brackets indicate the choroid layer (CH).

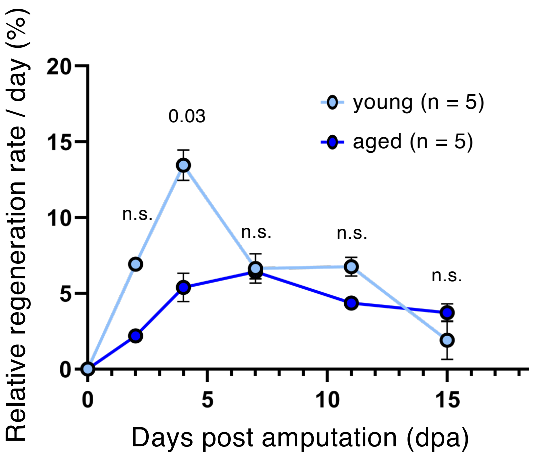

Supplementary Fig. 2: Aging leads to a decline in the fin regeneration rate.

The relative rate of fin regeneration per day in the young and aged fish from 0 to 15 dpa is shown (Mean ± SEM). An unpaired two-tailed *t*-test was used.

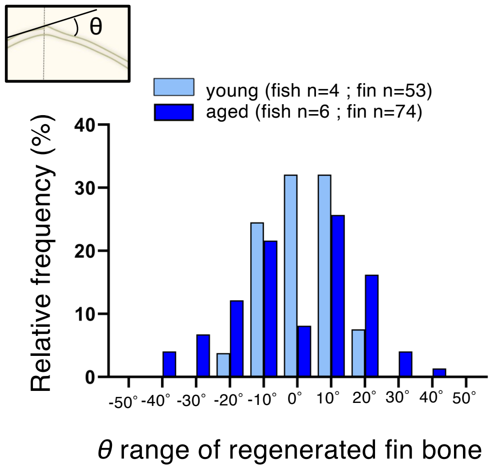

Supplementary Fig. 3: Aging increases misaligned fin regeneration.

Frequency histogram of the angle of regenerated fin bones at 15 dpa.

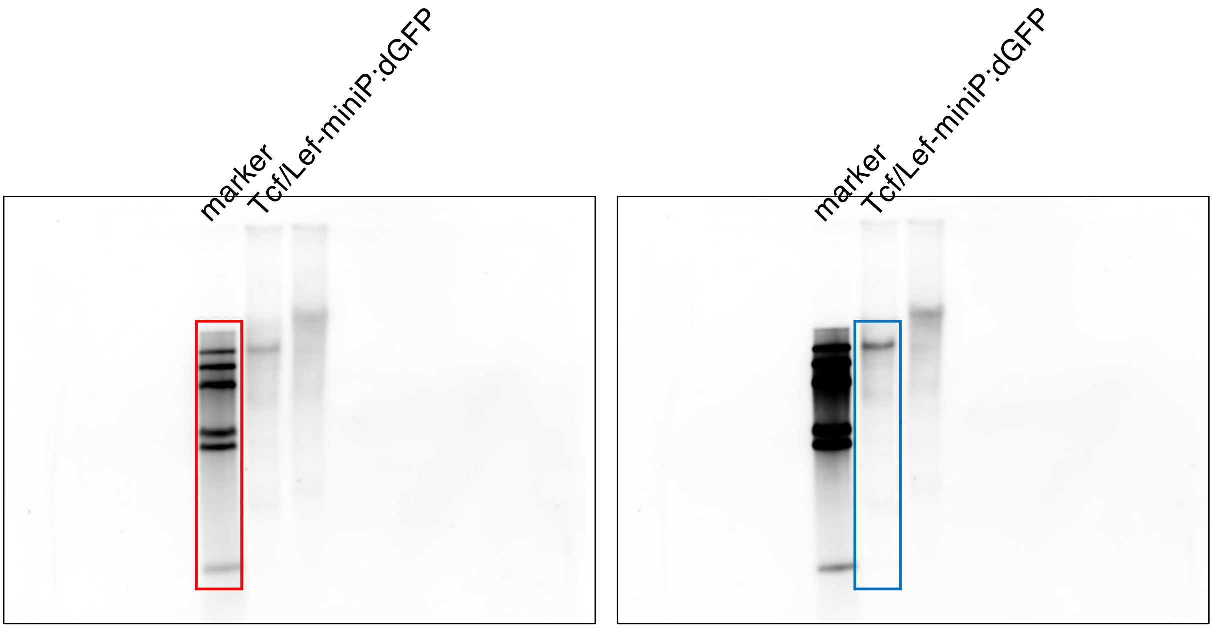

Supplementary Fig. 4 Uncropped scans of Southern blot in Fig. 1c. The cropped area are indicated red and frames. Left and right images were obtained from same membrane with short and long exposure, respectively.
